# Tree of motility – A proposed history of motility systems in the tree of life

**DOI:** 10.1111/gtc.12737

**Published:** 2020-01-19

**Authors:** Makoto Miyata, Robert C. Robinson, Taro Q. P. Uyeda, Yoshihiro Fukumori, Shun‐ichi Fukushima, Shin Haruta, Michio Homma, Kazuo Inaba, Masahiro Ito, Chikara Kaito, Kentaro Kato, Tsuyoshi Kenri, Yoshiaki Kinosita, Seiji Kojima, Tohru Minamino, Hiroyuki Mori, Shuichi Nakamura, Daisuke Nakane, Koji Nakayama, Masayoshi Nishiyama, Satoshi Shibata, Katsuya Shimabukuro, Masatada Tamakoshi, Azuma Taoka, Yosuke Tashiro, Isil Tulum, Hirofumi Wada, Ken‐ichi Wakabayashi

**Affiliations:** ^1^ Department of Biology Graduate School of Science Osaka City University Osaka Japan; ^2^ The OCU Advanced Research Institute for Natural Science and Technology (OCARINA) Osaka City University Osaka Japan; ^3^ Research Institute for Interdisciplinary Science Okayama University Okayama Japan; ^4^ School of Biomolecular Science and Engineering (BSE) Vidyasirimedhi Institute of Science and Technology (VISTEC) Rayong Thailand; ^5^ Department of Physics Faculty of Science and Technology Waseda University Tokyo Japan; ^6^ Faculty of Natural System Institute of Science and Engineering Kanazawa University Kanazawa Japan; ^7^ WPI Nano Life Science Institute (WPI-NanoLSI) Kanazawa University Kakuma-machi Kanazawa Japan; ^8^ Department of Biological Sciences Graduate School of Science and Engineering Tokyo Metropolitan University Tokyo Japan; ^9^ Division of Biological Science Graduate School of Science Nagoya University Nagoya Japan; ^10^ Shimoda Marine Research Center University of Tsukuba Shizuoka Japan; ^11^ Graduate School of Life Sciences Toyo University Gunma Japan; ^12^ Laboratory of Microbiology Graduate School of Pharmaceutical Sciences The University of Tokyo Tokyo Japan; ^13^ Laboratory of Sustainable Animal Environment Graduate School of Agricultural Science Tohoku University Miyagi Japan; ^14^ Laboratory of Mycoplasmas and Haemophilus Department of Bacteriology II National Institute of Infectious Diseases Tokyo Japan; ^15^ Department of Physics Oxford University Oxford UK; ^16^ Graduate School of Frontier Biosciences Osaka University Osaka Japan; ^17^ Institute for Frontier Life and Medical Sciences Kyoto University Kyoto Japan; ^18^ Department of Applied Physics Graduate School of Engineering Tohoku University Miyagi Japan; ^19^ Department of Physics Gakushuin University Tokyo Japan; ^20^ Department of Microbiology and Oral Infection Graduate School of Biomedical Sciences Nagasaki University Nagasaki Japan; ^21^ Department of Physics Faculty of Science and Engineering Kindai University Osaka Japan; ^22^ Molecular Cryo‐Electron Microscopy Unit Okinawa Institute of Science and Technology Graduate University Okinawa Japan; ^23^ Department of Chemical and Biological Engineering National Institute of Technology Ube College Yamaguchi Japan; ^24^ Department of Molecular Biology Tokyo University of Pharmacy and Life Sciences Tokyo Japan; ^25^ Department of Engineering Graduate School of Integrated Science and Technology Shizuoka University Shizuoka Japan; ^26^ Department of Botany Faculty of Science Istanbul University Istanbul Turkey; ^27^ Department of Physics Graduate School of Science and Engineering Ritsumeikan University Shiga Japan; ^28^ Laboratory for Chemistry and Life Science Institute of Innovative Research Tokyo Institute of Technology Kanagawa Japan

**Keywords:** appendage, cytoskeleton, flagella, membrane remodeling, Mollicutes, motor protein, peptidoglycan, three domains

## Abstract

Motility often plays a decisive role in the survival of species. Five systems of motility have been studied in depth: those propelled by bacterial flagella, eukaryotic actin polymerization and the eukaryotic motor proteins myosin, kinesin and dynein. However, many organisms exhibit surprisingly diverse motilities, and advances in genomics, molecular biology and imaging have showed that those motilities have inherently independent mechanisms. This makes defining the breadth of motility nontrivial, because novel motilities may be driven by unknown mechanisms. Here, we classify the known motilities based on the unique classes of movement‐producing protein architectures. Based on this criterion, the current total of independent motility systems stands at 18 types. In this perspective, we discuss these modes of motility relative to the latest phylogenetic Tree of Life and propose a history of motility. During the ~4 billion years since the emergence of life, motility arose in Bacteria with flagella and pili, and in Archaea with archaella. Newer modes of motility became possible in Eukarya with changes to the cell envelope. Presence or absence of a peptidoglycan layer, the acquisition of robust membrane dynamics, the enlargement of cells and environmental opportunities likely provided the context for the (co)evolution of novel types of motility.

## Graphical abstract

The known motilities are classified based on the unique classes of movement‐producing protein architectures. Based on this criterion, the current total of independent motility systems stands at 18 types. Presence or absence of a peptidoglycan layer, the acquisition of robust membrane dynamics, the enlargement of cells and environmental opportunities likely provided the context for the (co)evolution of novel types of motility.

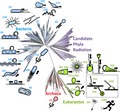

## 1 INTRODUCTION

**Figure 1:**
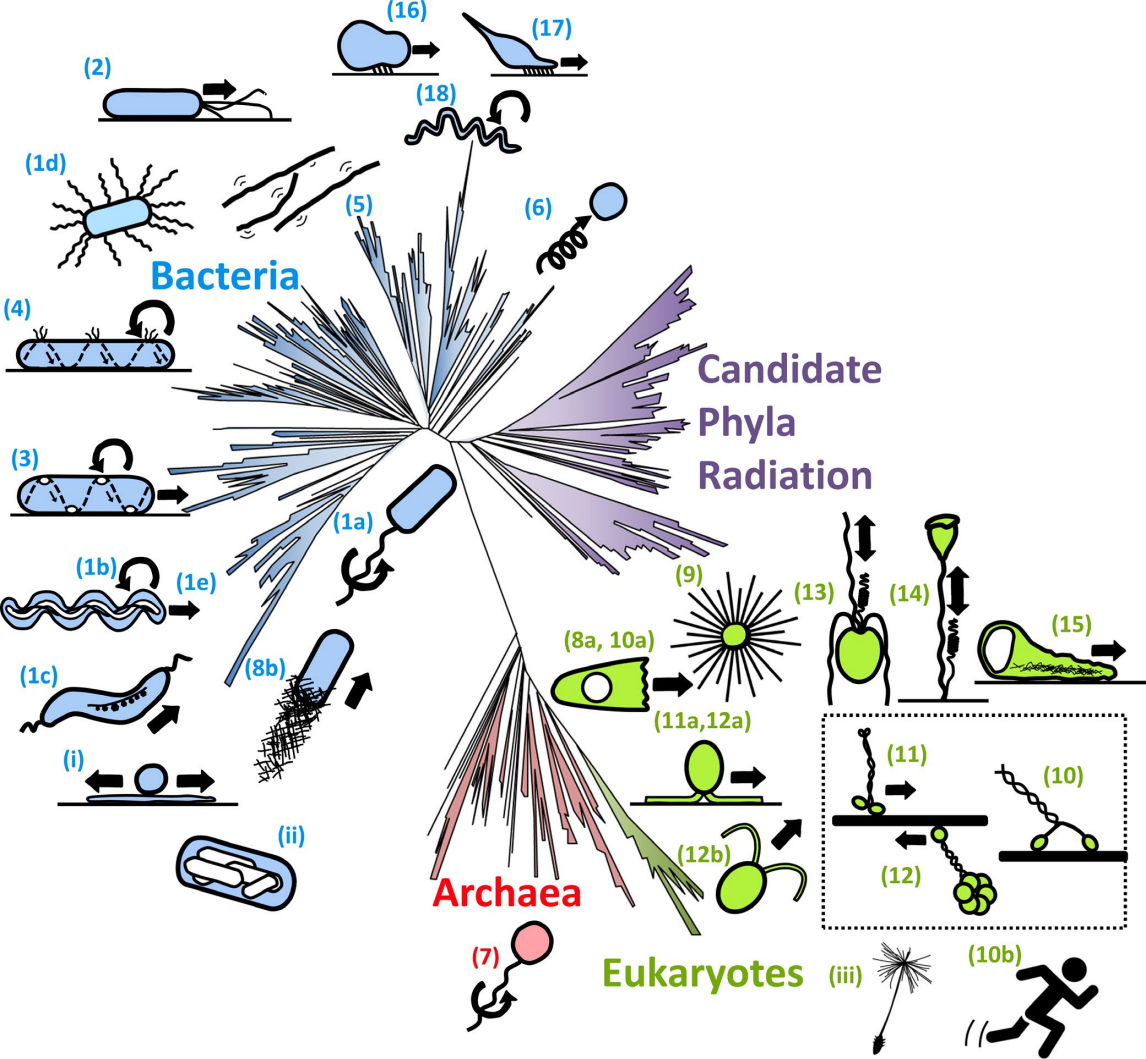
Various types of motility systems. Cartoons of those systems are listed according to the order in the text and roughly assigned to the relative positions in Tree of Life (Hug et al. 2016; Castelle & Banfield 2018). (1a) bacterial flagellar swimming, (1b) spirochetes flagellar swimming, (1c) magnetotactic bacterial flagellar swimming, (1d) bacterial flagellar swarming, (1e) *Leptospira* crawling motility, (2) bacterial pili motility, (3) *Myxococcus xanthus* adventurous (A) motility, (4) *Bacteroidetes gliding*, (5) *Chloroflexus aggregans* surface motility, (6) *Synechococcus* nonflagellar swimming, (7) archaella swimming, (8a) amoeba motility based on actin polymerization, (9) heliozoa motility based on microtubule depolymerization, (10) myosin sliding, (11) kinesin sliding, (12) dynein sliding, (10a) amoeba motility driven by contraction of cortical actin–myosin. (10b) animal muscle contraction, (11a, 12a) flagellar surface motility (FSM), (12b) flagellar swimming, (13) haptonemal contraction, (14) spasmoneme contraction, (15) amoeboid motility of nematode sperm, (8b) actin‐based comet tail bacterial motility, (16) *Mycoplasma mobile* gliding, (17) *Mycoplasma pneumoniae* gliding, (18) *Spiroplasma* swimming, (i) bacterial sliding, (ii) gas vesicle, (iii) dandelion seed. Refer to Table 1 for more details. The three eukaryotic conventional motor proteins are shown in the dotted box

Rapidly accumulating genomic data are changing our approaches to biology and our perspectives of the living organisms that inhabit this planet (Figure 1). According to the latest data analyses, life on the Earth can be divided into two or three groups (Brown et al., 2015; Castelle & Banfield, 2018; Hug et al., 2016; Williams, Foster, Cox, & Embley, 2013): Bacteria, which includes the subgroup of Candidate Phyla Radiation (CPR), species whose representatives have been confirmed to exist by microscopy or metagenomics but have yet to be cultured (Williams et al., 2013); Archaea; and Eukarya, a small group branching from Archaea to which *Homo sapiens* belongs. Archaea and Eukarya are grouped together in the two Domain hypothesis (Williams et al., 2013). The accumulating genomic data are useful to map the appearance of novel biological functions by tracing the presence of the encoding genes relative to branch points in the Tree of Life. In this perspective, we focus on the emergence of motility systems and propose a history of motility.

## 2 EIGHTEEN MOTILITY SYSTEMS

From the time of Leeuwenhoek, 350 years ago, people have been fascinated by motility, because movement is one defining feature of life (Berg, 2004). Motility can be a determinant for survival of species, by which living organisms obtain nutrients, escape from toxins and predators, and exchange genetic information through mating. It also plays critical roles in development and other physiological activities such as immune response and wound healing in multicellular organisms. Therefore, understanding the mechanisms of motility may provide useful information for controlling infectious microorganisms and benefit agriculture and medicine. Moreover, learning how protein motility machineries work may provide clues to develop artificial nanoscale actuators.

Today, the acquisition of genomic data coupled with advances in technologies in various fields such as genetic manipulation, structural analysis, imaging and single molecule measurements has enabled in‐depth investigation into motility. As a result, the mechanisms of many types of motility, which were previously regarded as mysteries, are now known at the molecular level. Although the types of locomotion of organisms are diverse, motility at the molecular level can be currently characterized as 18 distinct types of mechanism (Figure 1, Table 1). Here, "Motility" is defined as the ability of individual organisms or cells to convert chemical energy to locomotion of the whole organism or cell by using a dedicated motor system. Various kinds of criteria for classifying motion mechanisms are possible. We define a unique class of motility mechanism to have distinct structure of the force‐producing motor from any motor of another class of motility mechanism. According to these criteria, molecular movements such as those produced by rotary ATPases, helicases, DNA polymerases are not included as motility, because they do not propel a cell or organism. Similarly, movement of intracellular membrane vesicles driven by kinesin or dynein also does not qualify as motility. The current number of 18 types of motility is unlikely to be the final figure. In particular, CPR has yet to be explored from the motility perspective because of the intractability of cultivation, which leaves a systematic gap that likely conceals new mechanisms (Castelle & Banfield, 2018; Hug et al., 2016). In addition, there are examples of microorganisms that move immediately after isolation, yet become static after culture, which may hinder discovery of additional types of motility (Jishage & Ishihama, 1997). However, despite the advancing technology environment, no new type of motility has been discovered for more than ten years. Thus, the 18 types of motility mechanisms account for a substantial proportion of movement of observable organisms on Earth.

**Table 1:** Characterization of motility systems

Type	Name	Variations	Representatives	Key proteins	Energy source	Distribution	Year	References
1	Bacterial flagella swimming Spirochetes swimming	Typical flagella	(a) *Escherichia coli*	FliC, FliG, MotA, MotB	Proton motive force	Widespread in Bacteria	1974	Berg (1974), Larsen et al. (1974), Silverman and Simon (1974)
		Periplasmic flagella	(b) *Borrelia burgdorferi, Leptospira interrogans*	FlaB, FliG, MotA, MotB		Phylum Spirochaetes	1979	Li et al. (2000), Charon and Goldstein (2002), Charon et al. (2012), Takabe et al. (2017)
		Magnetotactic behavior	(c) *Magnetospirillum magneticum* AMB−1	FliC, FliG, MotA, MotB		Widespread in Bacteria	1975	Blakemore (1975), Blakemore, Maratea, and Wolfe (1979), Uebe and Schuler (2016)
		Swarming motility	(d) *Proteus mirabilis, Vibrio parahaemolyticus* (lateral flagella)	FliC, FliG, FliL, MotA, MotB		Widespread in Bacteria	1885	Henrichsen (1972)
		Crawling motility	(e) *Leptospira interrogans*	FlaB, FliG, MotA, MotB		*Leptospira*	1974	Cox and Twigg (1974), Tahara et al. (2018)
2	Bacterial pili motility		*Pseudomonas aeruginosae, Neisseria gonorrhoeae, Myxococcus xanthus* S motility *Synechocystis* sp. PCC6803	PilA, PilB, PilT	ATP	Widespread in Bacteria	1961	Lautrop (1961), Bradley (1972)
3	*Myxococcus* A motility		*Myxococcus xanthus*	AglRQS, GltA‐K	Proton motive force	Class Gammaproteobacteria		Nan et al. (2014), Agrebi et al. (2015), Mercier and Mignot (2016)
4	Bacterial gliding		*Flavobacterium johnsoniae*	SprB, GldBD, GldH‐*N*	Proton motive force	Phylum Bacteroidetes	1972	Pate and Chang (1979), Lapidus and Berg (1982), McBride and Nakane (2015)
5	Bacterial gliding		*Chloroflexus aurantiacus, Chloroflexus aggregans*	Unknown	Unknown	Phylum Chloroflexi	1995	Hanada, Hiraishi, Shimada, and Matsuura (1995), Hanada et al. (2002), Fukushima et al. (2016)
6	Bacterial swimming		*Synechococcus*	SwmA, SwmB	Unknown	*Synechococcus*	1985	Waterbury et al. (1985); Brahamsha (1999)
7	Archaella		*Halobacterium salinarum*	FlaH, FlaI, FlaJ	ATP	Phylum Euryarchaeota, Phylum Crenarchaeota	1984	Alam and Oesterhelt (1984), Jarrell and Albers (2012), Albers and Jarrell (2015), Chaudhury et al. (2016)
8	Actin polymerization‐based motility	(a) Amoeba motility driven by polymerization of actin filaments in pseudopods	Fish keratocytes, Leukocytes, *Dictyostelium discoideum*	Eukaryotic actin	ATP	Widespread in Animalia, Protist, Fungi	1993 (lamellipodia)	Tilney and Portnoy (1989), Theriot and Mitchison (1991), Small, Rohlfs, and Herzog (1993)
		(b) Comet tail motility of intracellular parasitic bacteria and virus	*Listeria monocytogenes*, Baculovirus			Bacteria, Virus	1989	Tilney and Portnoy (1989), Haglund and Welch (2011)
9	Microtubule depolymerization‐based motility		*Actinosphaerium nucleofilum, Actinocoryne contractilis, Echinosphaerium nucleofilum*, *Stentor polymorphus, Stentor coeruleus, Spirostomum ambiguum*	Tubulin	Ca^2+^ binding	Order Heliozoa, Class Heterotrichea	1965 1958	Randall and Jackson (1958), Tilney and Porter (1965), Ettienne (1970), Huang and Pitelka (1973), Suzaki et al. (1980), Febvre‐Chevalier and Febvre (1992)
10	Myosin sliding‐based motility	(a) Amoeboid motility driven by hydrostatic pressure	Metastatic cancer cells, *Amoeba proteus, Physarum polycephalum, Dictyostelium discoideum*	Actin and myosin II	ATP	Widespread in Animalia, Protist, Fungi	1984 (contraction of the rear of amoeboid cells)	Yumura, Mori, and Fukui (1984), Bray and White (1988)
		(b) Muscle contraction	Striated muscle, Jelly fish	Actin and myosin II	ATP	Widespread in Animalia	1954 (striated muscle)	Szent‐Györgyi (1947), Huxley and Niedergerke (1954), Huxley and Hanson (1954), Seipel and Schmid (2005)
		(c) Various motilities driven by unconventional myosin moving along actin filaments	*Toxoplasma gondii*, *Bacillaria paxillifer*	Actin and unconventional myosin	ATP	Sporadic in Protist		Meissner, Schluter, and Soldati (2002), Yamaoka, Suetomo, Yoshihisa, and Sonobe (2016)
11	Kinesin sliding‐based motility	Vesicle transport (not motility)	Wide range of eukaryotes	Kinesin, Tubulin	ATP	Widespread in eukaryotes	1985	Bloodgood, Leffler, and Bojczuk (1979), Vale, Reese, and Sheetz (1985), Shih et al. (2013)
		(a) Flagellar surface motility	*Chlamydomonas reinhardtii*			Protist, Animalia	1979	Bloodgood et al. (1979), Shih et al. (2013)
12	Dynein sliding‐based motility	(b) Eukaryotic ciliary and flagellar swimming	Wide range of eukaryotes other than Plantae	Dynein, Tubulin	ATP	Widespread in eukaryotes other than Plantae	1972	Gibbons and Gibbons (1972), Gibbons and Fronk (1972), Iyer et al. (2004), Shih et al. (2013)
		(a) Flagellar surface motility	*Chlamydomonas reinhardtii*					Bloodgood et al. (1979), Shih et al. (2013)
13	Haptonema coiling		*Chrysochromulina acantha, Chrysochromulina simplex, Chrysochromulina hirta*	Tubulin	Ca^2+^ binding	Class Haptophyceae	1955	Parke et al. (1955), Greyson et al. (1993), Kawachi and Inouye (1994)
14	Spasmoneme coiling		*Vorticella convallaria, Zoothamnium geniculatum, Carchesium polypinum*	Spasmin	Ca^2+^ binding	Subclass Peritrichia	1958	Hoffman‐Berling (1958), Amos et al. (1975)
15	Amoeboid motility of nematode sperm		*Caenorhabditis elegans*, *Ascaris suum*	Major sperm protein (MSP)	ATP	Phylum Nematoda	1979	Roberts and Stewart (1997)
16	*Mycoplasma* gliding		*Mycoplasma mobile*	Gli349, Gli521	ATP	Class Mollicutes	1977	Miyata (2010), Miyata and Hamaguchi (2016b)
17	*Mycoplasma* gliding		*Mycoplasma pneumoniae*	P1 adhesin, HMW2	ATP	Class Mollicutes	1968	Bredt (1968), Miyata and Hamaguchi (2016a), Mizutani and Miyata (2019)
18	*Spiroplasma* swimming		*Spiroplasma melliferum*	Fib, MreB	Unknown	Class Mollicutes	1973	Wada and Netz (2009), Liu et al. (2017)
*Motility not driven by dedicated motor system*								
(i)	Sliding motility		*Bacillus subtilis*	Surfactin	Surface tension	Phylum Firmicutes, Phylum Actinobacteria	1972	Martinez, Torello, and Kolter (1999)
(ii)	Gas vesicle floating		*Halobacterium salinarum*,* Anabaena flos‐aquae*,* Serratia* sp.	GvpA	Buoyant force	Photosynthetic bacteria, Haloarchaea, Heterotrophic bacteria	1895	Pfeifer (2012), Tashiro et al. (2016)
(iii)	Plant seed flying		Dandelion, maple	NA	Air flow	Widespread in higher plants	NA	

The 18 types of motility are numerous when compared to the five conventional types of motility: those driven by bacterial flagella; by the eukaryotic motor proteins myosin, dynein or kinesin; or by actin polymerization. Nonetheless, taking into account that the evolution of life has been ongoing for ~4 billion years, it appears that novel motility mechanisms only sporadically evolve. It is also noteworthy that different modes of motility have evolved, considering that other vital processes, such as the mechanisms for ATP synthesis (Gogarten & Taiz, 1992) and protein synthesis (Yao & O'Donnell, 2016), have retained core machineries. This is probably because motility is established by the interaction of the organism with its environment. Organisms and environments have been constantly changing during the ~4 billion years of life. Conditions underwater, on a wet surface, in the bacterial flora, in hard soil or in tissues of hosts likely required different mechanisms for effective locomotion. Clear examples of adaptation to changing conditions are observed in host–pathogen interactions (see the section on class Mollicutes below). The appearance of a novel motility of a pathogen in a host would only be possible, at the earliest, during the emergence of the host. Furthermore, the architecture of a cellular motility machine needs to couple the motor output with the physical properties of cell envelope in order to produce sufficient force to propel the whole‐cell body via interactions with its environment. This equation has been solved in different ways by organisms in various branches of the Tree of Life and appears to be critically dependent on the nature of the cell envelope. The order of events of how a mechanism of motility evolved, and how it may have ceased to function to be replaced by a new mode of motility, has yet to be temporally delineated with respect to the evolution of life on Earth. In the following sections, we propose a history of movement of life on Earth, which differs from the Tree of Life (Castelle & Banfield, 2018; Hug et al., 2016), in accordance with the order in which motility mechanisms arose, based on the latest understanding from the multiple types of motility and genomic data.

## 3 BACTERIA

Many Bacteria move based on manipulating external appendages, by swimming using flagella (Figure 1; type 1a) or by the shortening of pili (Figure 1; type 2) (Berg, 1974, 2003; Larsen, Reader, Kort, Tso, & Adler, 1974; Lautrop, 1961; Mattick, 2002; Silverman & Simon, 1974). Motilities that depend on flagella and pili are widely distributed in many systematically separated phyla and their broad distributions suggest that these types of motility are robust and adaptable (Berry & Pelicic, 2015; Pallen, Penn, & Chaudhuri, 2005). Indeed, there are many variations in flagella‐powered swimming, such as spirochetes swimming (Figure 1; type 1b) (Charon & Goldstein, 2002; Li, Motaleb, Sal, Goldstein, & Charon, 2000), surface movement (Figure 1; type 1d) (Harshey & Partridge, 2015; Kearns, 2013; Patrick & Kearns, 2012) called swarming and swimming in response to geomagnetism sensed by magnetotactic bacteria (Figure 1; type 1c) (Blakemore, 1975; Uebe & Schuler, 2016). In addition, differences are observed in the ions used for torque generation as adaptations to the environment (Ito & Takahashi, 2017).

It is thought that these motility mechanisms are widely distributed because flagella and pili existed from an early stage of Bacteria evolution or were spread due to horizontal gene propagation, scenarios that are difficult to distinguish (Pallen & Matzke, 2006; Pallen et al., 2005). Probably, both routes occurred. However, it is worth noting that flagellar movement is also observed in the deeply branching Bacteria, such as *Aquificaceae* (Takekawa et al., 2015) and *Thermotogae* (Liu & Ochman, 2007). Bacterial flagella are held in the cell envelope at multiple places for high‐speed motor rotation (Chang et al., 2016; Minamino & Imada, 2015; Minamino, Imada, & Namba, 2008). In *E. coli* and *Salmonella*, flagella interact with the cell envelope through three basal body rings (MS, L and P rings) and stators. Thus, one of the principles of motility mechanisms in single cells is the compatibility with the cell envelope architecture. Flagella and pili are complicated systems composed of many proteins and it is difficult to trace the nearest ancestors whose structures are similar but have different roles (Pallen & Matzke, 2006; Pallen et al., 2005). Similarities in constituent proteins suggest that the basal body that rotates flagella and the basal body that expands/contracts pili have the same molecular origin as the transporter that transfers proteins across a cell membrane (Minamino, 2014; Mulkidjanian, Makarova, Galperin, & Koonin, 2007; Rapoport, Li, & Park, 2017). Flagella and pili are equipped with protein transporting abilities, but there are many other protein transporting devices that have not evolved into motility machines. Thus, a flagella/pili‐related transport device likely occurred in the earliest Bacteria, and this system was later duplicated and adapted to engender motility.

Some Bacteria show a specialized motility resulting from the movement of small structures on the surface of the cell, much smaller than the large appendages of flagella and pili. These bacteria travel in high‐viscosity environments such as soil, microbial mats, host tissues and three‐dimensional intergrowths. These new motilities likely evolved because of the excessive amount of force needed to move large appendages, such as flagella and pili, in these confined environments. This is the case for some Proteobacteria (Agrebi, Wartel, Brochier‐Armanet, & Mignot, 2015; Mercier & Mignot, 2016; Nan, McBride, Chen, Zusman, & Oster, 2014) typified by the A motility of *Myxococcus xanthus* (Figure 1; type 3) (Agrebi et al., 2015; Mercier & Mignot, 2016; Nan et al., 2014), the gliding motility of Bacteroidetes (Figure 1; type 4) (McBride & Nakane, 2015; Nakane, Sato, Wada, McBride, & Nakayama, 2013; Wada, Nakane, & Chen, 2013) represented by *Flavobacterium johnsoniae*, and the surface motion of thermophilic filamentous bacteria *Chloroflexus aggregans* classified as phylum Chloroflexi (Figure 1; type 5) (Fukushima, Morohoshi, Hanada, Matsuura, & Haruta, 2016; Hanada, Shimada, & Matsuura, 2002). Spirochete swimming is a variation of the flagellar swimming described above, but achieves a smooth motion by placing flagella inside the outer membrane, in the periplasmic space (Figure 1; type 1b) (Charon et al., 2012; Charon & Goldstein, 2002; Li et al., 2000; Takabe, Kawamoto, Tahara, Kudo, & Nakamura, 2017). The motility of *Myxococcus xanthus* (phylum Proteobacteria, Figure 1; type 3) and the gliding movement of *Flavobacterium johnsoniae* (phylum Bacteroidetes, Figure 1; type 4) are well studied. These two phyla are not systematically close in evolution, and as there is no significant amino acid sequence homology between the proteins producing the motility, it is probable that their motility systems occurred independently. However, it is interesting that both of these protein complexes transmit force to the substrate surfaces by moving a helix on the periphery of their respective bacterial cells, using membrane potential, electrochemical potential to be exact, as the energy source.

It has long been known that filamentous cyanobacteria perform surface motions, and that these movements result from type IV pili (Duggan, Gottardello, & Adams, 2007; Khayatan, Meeks, & Risser, 2015; Wilde & Mullineaux, 2015). Additionally, *Synechococcus*, a marine cyanobacteria, is known to swim at a speed of 25 μm/ s by a mechanism different to that of bacterial flagella (Figure 1; type 6) (Waterbury, Willey, Franks, Valois, & Watson, 1985). Formation of waves on the cyanobacteria surface is thought to push surrounding water backwards (Ehlers & Oster, 2012). These four types of motility (Figure 1; types 3–6) are limited to each phylum, suggesting that they arose relatively later in the evolution of Bacteria. Interestingly, all of these phylum‐specific movements are found in Gram‐negative bacteria. This may be due to the possibility to create new mechanisms in the periplasmic space specific to Gram‐negative bacteria or that the Gram‐positive bacteria peptidoglycan layer is more rigid and difficult to adapt for movement. In the evolution of bacterial motility, viscosity of surrounding media and interaction with environmental surfaces would have been critical factors. To this end, some bacterial species are equipped with dual flagellar systems, one used for swimming (a constitutive polar flagellum) and the other used for swarming on the surfaces (inducible lateral flagella; McCarter, 2004). Viscosity appears to be sensed by the flagellar motor as the environmental load, and cells adjust the power output of the motor by changing the number of energy converting units (Lele, Hosu, & Berg, 2013; Minamino, Terahara, Kojima, & Namba, 2018; Nord et al., 2017). Environmental conditions are significant factors in a low Reynolds number world, in which small cells, such as bacteria, are influenced more by friction with surrounding subjects rather than the inertia caused by their masses.

In CPR genomes, genes homologous to those for bacterial flagella and pili are found, suggesting the wide distribution of these common motility systems (Nelson & Stegen, 2015). However, genes involved in other bacterial motility systems, such as pili‐independent gliding, *Synechococcus* swimming and Mollicutes motility, are not found in the current CPR genomes. Discovery of novel, CPR‐specific motilities will likely become possible only after those organisms are cultured.

## 4 ARCHAEA

Many phyla of Archaea swim with “flagella” called archaella (Figure 1; type 7) (Jarrell & Albers, 2012). The structure of archaella has nothing in common with bacterial flagella besides the gross overall shape; rather, they share similarity in some protein components to, and likely evolved from, bacterial pili. Interestingly, other classes of motility identified in Bacteria (Figure 1; types 1–6) have not been found in Archaea to date. Thus, it appears that bacterial motility systems were not successfully transferred to Archaea even though some archaea inhabit common environments with bacteria. This may be related to the fact that Archaea do not have a peptidoglycan layer (Albers & Meyer, 2011; Daum et al., 2017), although most bacterial motility systems, including flagella (Minamino & Imada, 2015; Minamino et al., 2008) and pili (Shahapure, Driessen, Haurat, Albers, & Dame, 2014), depend mechanistically on anchoring to the peptidoglycan layer. This poses the question: How does the archaellum rotate without a peptidoglycan layer for support? It is anchored at a single position via a protein that binds to the pericellular S‐layer (Banerjee et al., 2015), the outermost layer of Archaea that often consists of paracrystalline arrays of a single or small number of proteins or glycoproteins. In bacterial pili, an ATPase hexamer rotates at the base of the pilus, which is responsible for pili extension and retraction (Chang et al., 2016; McCallum, Tammam, Khan, Burrows, & Howell, 2017). This rotational property appears to have been adapted in archaella to be used for swimming. It is interesting that the Archaea swimming mode resembles the bacterial flagella swimming mode (Kinosita, Uchida, Nakane, & Nishizaka, 2016), because it likely evolved from pili that display a different mode of motility. Probably, this is the result of the optimal motility format being dictated by physical factors such as cell size, viscosity of water, required swim speed, and protein stiffness (Magariyama et al., 1995; Wada & Netz, 2007). Thus, flagella and archaella are examples of convergent evolution, similar to the convergent evolution of the motilities of *Myxococcus xanthus* A and *Flavobacterium johnsoniae* arising from different machineries, as well as to the two types of gliding motility found in Mollicutes, discussed later (Miyata & Hamaguchi, 2016a, 2016b). To date, archaella are the sole identified motility system in archaea; however, we cannot rule out the existence of other systems, because the detailed analyses of archaea behavior started relatively recently in comparison with the studies of bacteria (Kinosita et al., 2016).

## 5 EUKARYA

Archaea have a proteinaceous S‐layer which likely lacks some of the mechanical strength of the bacterial peptidoglycan layer. It is speculated, from gene homology, that an Asgard‐like archaeon acquired membrane dynamics and enlarged its cell and genome sizes to evolve to Eukarya (Akil & Robinson, 2018; Spang et al., 2015; Zaremba‐Niedzwiedzka et al., 2017). Metagenome analyses have showed that Asgard archaea genomes, including Lokiarchaeota, contain potential genes for eukaryote‐like membrane fusion, membrane distortion and secretion machineries (Spang et al., 2015; Zaremba‐Niedzwiedzka et al., 2017). During the evolution of eukaryotes, cell enlargement and the availability of enhanced energy sources (from mitochondria, chloroplasts or phagocytosis) alleviated some of the constraints on protein machineries (Lane & Martin, 2010). In addition, because it became possible to support larger genomes, the expansion of the total DNA allowed for encoding of proteins for new functions. However, one ramification of increasing cell size during the archaea‐to‐eukaryote transition would be difficulty in moving using the existing motility mechanisms. Furthermore, concurrently with cell expansion, it became necessary to actively transport materials within the cytoplasm, something that could be left to diffusion in smaller cells (Koch, 1996).

In Bacteria, the process of diffusion is sufficient for mass transport for most substances. This situation is true for the bacterial genus *Thiomargarita*, whose diameter reaches 750 μm, because these giant bacteria are polynuclear and their cytoplasm is thin, which minimizes the distances and volumes for transport (Schulz, 2006). The movement of larger structures, such as the arrangement of peptidoglycan synthase (Busiek & Margolin, 2015), DNA distribution in plasmid partitioning (Popp & Robinson, 2011; Salje, Gayathri, & Löwe, 2010), and cell division, are performed in Bacteria by the polymerization and depolymerization of MreB, ParM and FtsZ, which share ancestors with eukaryotic actin and tubulin. Asgard archaea, or early Eukarya, can be speculated to have used polymerization to organize cell membranes, membrane vesicles, cytoplasm and chromosomes (Makarova, Yutin, Bell, & Koonin, 2010; Spang et al., 2015; Wickstead & Gull, 2011; Zaremba‐Niedzwiedzka et al., 2017). In particular, the movement of the membranes by actin polymerization, which is structurally related to MreB and ParM, led to the acquisition of a new motility, amoeboid movement (Figure 1; type 8a). Amoeboid motility is closely related to phagocytosis. The ability to internalize other cells increased the efficiency of food uptake when compared with energy‐dependent uptake of molecular nutrients across the cell membrane from the dilute surrounding medium. A similar amoeboid motility is observed also for tubulin polymerization in order Heliozoa and class Heterotrichea.

More efficient transport systems using the cytoskeleton have evolved through developing “conventional” motor proteins, such as myosin, kinesin and dynein, which move along actin filaments and microtubules (Figure 1; types 10–12). Myosin and kinesin are related to each other, and moreover, all are classified to the P‐loop NTPases. Although the prototypic motor protein cannot be traced from their extant structures, it should be emphasized that a number of nonmotor proteins in their class, including translation elongation factors, helicases, proteasomes, are known to generate force. These proteins may be related to the conventional motor protein at the root of cellular evolution (Iyer, Leipe, Koonin, & Aravind, 2004; Kull, Vale, & Fletterick, 1998; Leipe, Wolf, Koonin, & Aravind, 2002; Vale & Milligan, 2000). The class II myosin that forms bipolar filaments emerged after the unconventional myosin had evolved as a transporter. Interaction of bipolar myosin II filaments and actin filaments enabled a new mode of motility; contraction. Contraction drives muscle force generation (Figure 1; type 10b) as well as contributing to amoeboid movement that is also dependent on the contraction of actin and myosin II underneath the cell membrane (Figure 1; type 10a) (Paluch & Raz, 2013; Wessels et al., 1988). Contraction also enabled efficient cytokinesis in cells by forming contractile rings (Uyeda & Nagasaki, 2004), aiding the development of multicellular organisms. In the similar way, the interactions of dynein with microtubules drive movements of eukaryotic flagella for swimming (Figure 1; type 12b) (Gibbons, 1963).

As many Eukarya are soft cells, movements within a cell can be transmitted to the outside. When this was advantageous for survival, new modes of motility had the opportunity to arise. The scenario that the movement of a transport system gave rise to a new motility can be observed in the form of protozoan flagella and cilia, called flagellar surface motility (FSM; Figure 1; types 11a,12a; Shih et al., 2013). This is the ability of the flagella and cilia to glide on a solid surface which is caused by the transport of membrane vesicles in flagella and cilia, that is, intraflagellar transport (IFT). In this system, kinesin and dynein are directly involved in cell migration.

Some unicellular eukaryotes show other unique examples that transmit motion from inside of a eukaryotic cell to the exterior to produce atypical motilities. The haptonema (Figure 1; type 13) (Greyson, Green, & Leadbetter, 1993; Kawachi & Inouye, 1994; Nomura et al., 2019; Parke, Manton, & Clarke, 1955), a filiform structure in haptophytes, is rapidly coiled by mechanical stimuli through Ca^2+^‐dependent changes of microtubule configurations. A morphologically similar structure, the spasmoneme (Figure 1; type 14) (Amos, Routledge, & Yew, 1975; Hoffman‐Berling, 1958), in peritrichous ciliates contracts by the structural changes of Ca^2+^‐binding protein spasmin in a microtubule‐independent manner. Axopodia and stalk (Febvre‐Chevalier & Febvre, 1992; Suzaki, Shigenaka, Watanabe, & Toyohara, 1980; Tilney & Porter, 1965) in heliozoans show rapid contractions caused by catastrophic microtubule breakdown. Heterotrichous ciliates use a combination of microtubules and actin‐like filaments to create large deformations of their cell bodies for movement (Huang & Pitelka, 1973; Randall & Jackson, 1958; Tilney & Porter, 1965). In nematode sperm (Figure 1; type 15), the treadmilling polymerization of the unique protein MSP drives forward its amoeboid cell body, in a manner similar to actin‐driven amoeboid movement (Roberts & Stewart, 1997).

## 6 ACTIN‐BASED MOTILITY BY PARASITIC BACTERIA

The emergence of large Eukarya cells with flexible membranes and intracellular transport systems provided the opportunity for Bacteria to invade these environments (Carayol & Tran Van Nhieu, 2013; Dey & Bishai, 2014; LaRock, Chaudhary, & Miller, 2015; McFadden, 2014; Pizarro‐Cerda, Kuhbacher, & Cossart, 2012). Such Bacteria often hijack membrane fusion, phagocytosis and cytoskeleton machineries of the host Eukarya. *Shigella* and *Listeria* enter the eukaryotic cells and induce actin polymerization of the host cell by presenting mimetics of the eukaryotic actin regulating proteins at one end of the Bacteria, forming an actin comet tail to move inside the host cell and spread to the neighboring host cells (Figure 1; type 8b) (Stevens, Galyov, & Stevens, 2006; Tilney & Portnoy, 1989; Yoshida et al., 2006).

## 7 BACTERIAL MOLLICUTES

A small group of organisms, class Mollicutes, which has evolved from the phylum Firmicutes including *Bacillus* and *Clostridium*, have acquired three distinct motility mechanisms (Miyata & Hamaguchi, 2016a). These are the gliding motility (Bredt & Radestock, 1977; Miyata, 2010; Miyata & Hamaguchi, 2016b) displayed by *Mycoplasma mobile,* which is a pathogen of freshwater fish, a second type of gliding motility (Figure 1; type 16) (Bredt, 1968; Miyata & Hamaguchi, 2016a) used by *Mycoplasma pneumoniae* in humans (Figure 1; type 17), and the swimming motility of *Spiroplasma* (Figure 1; type 18) (Cole, Tully, Popkin, & Bove, 1973; Liu et al., 2017; Shaevitz, Lee, & Fletcher, 2005; Wada & Netz, 2009), a common pathogen of plants and arthropods.

One reason why as many as three motility mechanisms are acquired in a small class may be related to class Mollicutes abandoning the peptidoglycan layer as in the case of Eukarya. The loss of peptidoglycan is likely related to the parasitic or commensal lifestyle of Mollicutes. Generally, in order for Bacteria to live inside animals and plants, it is necessary to deal with the stress of peptidoglycan layer decomposition by lysozyme (Kawai, Mickiewicz, & Errington, 2018; Tulum, Tahara, & Miyata, 2019). Also, peptidoglycans are a target of innate immunity (Royet & Dziarski, 2007; Royet, Gupta, & Dziarski, 2011). By dispensing with the peptidoglycan synthesis system, the organism is relieved from these stresses and furthermore can reduce its requirements for cellular materials, energy and genomic size. The cells become smaller and softer, which may also help in concealment in the host. Class Mollicutes appears to have relinquished this peptidoglycan layer synthesis as a survival strategy. At the same time, like Archaea, the bacterial flagellum could not function in class Mollicutes, in the absence of a peptidoglycan layer. However, for class Mollicutes to live in host–animal tissue, motility is probably an important strategy for survival, in the processes of infection and evading cellular immune systems. As cells of class Mollicutes are small, unlike Eukarya, intracellular transport devices are not required, but since they have a soft peripheral structure, it would be relatively simple to convey the movement of housekeeping activities present in cells to the outside.

Among housekeeping activities other than intracellular transport, there are many that involve movements of nanometer scale. These include nucleic acid polymerization [RNA polymerase (Gelles & Landick, 1998); helicase (Tuteja & Tuteja, 2004)], protein synthesis (Rodnina, Savelsbergh, & Wintermeyer, 1999), ATP synthesis (Noji, Yasuda, Yoshida, & Kinosita, 1997; Oster & Wang, 1999), protein secretion (Goldman et al., 2015; Ismail, Hedman, Schiller, & Heijne, 2012; Ito & Chiba, 2013), DNA partitioning [eukaryotic chromosome (Vernos & Karsenti, 1996), plasmid segregation (Salje et al., 2010)] and cell division (Mabuchi & Okuno, 1977; Rappaport, 1971). In fact, the gliding motility mechanism of *M. mobile* is thought to originate from an altered ATP synthase, which has been coupled to an adhesin, and the swimming ability of *Spiroplasma* is thought to originate from the structural changes in the cytoskeleton used for synthesis of peptidoglycan layer and segregation of DNA. The fact that most of class Mollicutes isolated so far rely on higher animals and plants for their survival suggests that these types of motility arose after the Cambrian explosion (Miyata & Hamaguchi, 2016a).

## 8 “MOTILITY” NOT DIRECTLY LINKED TO ENERGY

In this article, motions that are driven by mechanisms other than dedicated motor systems are not included in our classification of motility, but there are many examples of movement that fall outside this classification. Examples are found in Bacteria such as the colony spreading of *Staphylococcus aureus* (Figure 1; type i) and the sliding motility of *Bacillus subtilis* (Holscher & Kovacs, 2017). These bacteria can spread on a solid medium by the spreading force in cell division under the control of surface tension through secreting substances that act as surfactants around the cells (Henrichsen, 1972; Kearns, 2010). Another example is the gas vesicles formed in cells by microorganisms present in the hydrosphere such as the phylum Cyanobacteria and Haloarchaea, which move up and down in the environment using the gas vesicles akin to a fish bladder (Figure 1; type ii) (Tashiro, Monson, Ramsay, & Salmond, 2016; Tavlaridou, Winter, & Pfeifer, 2014). "Motility" which does not link directly with energy consumption is known in many higher plants, which have hard cell walls and are difficult to move. For example, turgor pressure in seed pods of violets or the drying of pea pods that can mechanically dissipate the seeds, and the wind dispersal of maple and dandelion seeds (Figure 1; type iii), which could be included in a less strict definition of motility.

## 9 CONCLUSIONS

In this perspective, we performed an initial tracing of the evolution of motility systems to produce a Tree of Motility, in which the peptidoglycan layer and the emergence of large cells play critical roles. In order to make this “Tree” more complete and exact, the following information will be useful: (a) quantitative mapping on the phylogenic tree, based on the structural features of the proteins responsible for each motility, (b) more genome information to fill the gaps between different systems, (c) elucidation of each motility mechanism, (d) discovery of new modes of motility particularly those in CPR, and (e) model experiments reproducing evolution. Thus, this perspective represents the first step in cataloging and establishing the history of emergence of the modes of motility on this planet.

## CONFLICT OF INTEREST

There are no conflicts of interest to declare.
